# Effects of a Comprehensive Pulmonary Rehabilitation in Severe Post-COVID-19 Patients

**DOI:** 10.3390/ijerph18052695

**Published:** 2021-03-07

**Authors:** Marc Spielmanns, Anna-Maria Pekacka-Egli, Sabine Schoendorf, Wolfram Windisch, Matthias Hermann

**Affiliations:** 1 Pulmonary Medicine and Sleep Medicine Center, Zurich RehaCenter Klinik Wald, CH-8636 Wald, Switzerland; annamaria.pekacka@zhreha.ch (A.-M.P.-E.); sabine.schoendorf@zhreha.ch (S.S.); 2 Department of Pulmonary Medicine, Faculty of Health, University Witten-Herdecke, D-58455 Witten, Germany; windischw@kliniken-koeln.de; 3 Neurological Rehabilitation, Zurich RehaCenter Klinik Wald, CH-8636 Wald, Switzerland; 4 Department of Pneumology, Cologne Merheim Hospital, Kliniken der Stadt Koeln GmbH, D-51109 Koeln, Germany; 5 Department of Cardiology, University Heart Centre, University Hospital Zurich, CH-8006 Zurich, Switzerland; matthias.hermann@usz.ch

**Keywords:** COVID-19, pulmonary rehabilitation, FIM, 6-MWT, FT, CIRS

## Abstract

Background: Severe COVID-19 infection often leads to impairments requiring pulmonary rehabilitation (PR) following the acute phase. Little is known about the efficacy of PR in these patients. We therefore compared post-COVID-19 patients (PG) referred to PR patients with other lung diseases (LG). Methods: 99 PG were admitted to PR. In a prospective design, the results of PG were collected and compared to the results of LG of 2019 (*n* = 419) according to Functional Independence Measurement (FIM), Cumulative Illness Rating Scale (CIRS), 6-min walk test (6-MWT), duration of PR, and Feeling Thermometer (FT). Results: According to age, sex, and CIRS, both groups showed no significant differences. The improvements in the 6-MWT in the pre to post comparison were on average 180 (±101) meters for PG and 102 (±89) meters for LG (*p* < 0.001). FT showed a significant enhancement for PG of 21 (±14) points and for LG of 17 (±16) points (*p* < 0.039), while FIM significantly increased by 11 (±10) points in PG and 7 (±8) points in LG (*p* < 0.001). Conclusions: Comprehensive PR in PG is very effective according to the results in FIM, 6-MWT and FT. Therefore, we recommend PR following severe post-COVID-19 infections.

## 1. Introduction

The COVID-19 pandemic was first identified in Wuhan, Hubei Province, China, in December 2019 [1]. Patients with COVID-19 typically present with symptoms of fever, cough, and dyspnea [2,3,4]. Less common symptoms include myalgias/fatigue, rhinorrhea, sore throat, headache, and diarrhea [2]. Bilateral ground glass opacities, as well as bilateral multiple lobular and subsegmental areas of consolidation, are described as typical imaging findings [5]. Complications include respiratory failure, non-pulmonary complications such as acute myocardial injury, renal failure or thromboembolic events, septic shock, and multiple organ failure in severe cases. Therefore, a large number of patients with severe COVID-19 suffer from functional physical and partly psychological limitations [6,7].

A significant number of patients with COVID-19 admitted to rehabilitation have spent time in the intensive care unit (ICU) and have symptoms common to other ICU patients, including dyspnea, anxiety, depression, prolonged pain, impaired physical function and poor quality of life [7,8]. Although the lung is the primary target of coronavirus infection, clinical signs of central (i.e., dizziness, headache, and/or impaired consciousness) and/or peripheral nervous system (i.e., taste and/or smell impairment) involvement have been reported, especially in patients requiring mechanical ventilation [9].

Based on the above-described functional deficits, many patients need pulmonary rehabilitation (PR) following the acute phase. For these patients, a step-by-step concept of a comprehensive multimodal and interdisciplinary PR is needed, differentiated according to the extent of the impairment caused by the infection, the activity restrictions, and the participation disorders. For most patients, an inpatient setting is necessary due to the complexity and degree of the limitations [10]. However, inpatient PR during the COVID-19 pandemic can only occur under considerable restrictions (significantly reduced number of patients and therapies, personnel–intensive care, high hygienic expenses), especially if isolation is still required. In a previous study, we showed that isolation not only leads to reduction of therapies but also has a negative impact on the outcome of a pulmonary rehabilitation program [11].

The course of the disease can be complicated by the necessity of retransfer to an acute clinic due to a renewed deterioration. Many patients admitted to PR still have residual infiltrates and/or possibly permanent fibrosis/interstitial affections causing limitations in gas exchange [12]. A close monitoring of the course containing functional diagnostics and imaging of these changes is required, especially with regard to the resulting functional restrictions [13,14].

The goals of PR in COVID-19 are very similar to those of PR in other pulmonary diseases, i.e., improvements in persisting functional physical limitations, performance, endurance, and disabilities resulting from further organ complications. According to the psychological aspects, professional support in coping with the illness after an often long and complicated intensive stay is necessary [15]. Finally, restoration of the ability to perform at work and in everyday social life represents the participation-oriented goals. However, little is known regarding to what extent these goals of PR can be achieved in post-COVID-19 patients, particularly in comparison to patients with pulmonary diseases usually referred to PR. The first experiences with PR in post-COVID-19 patients indicated that improvements were significant according to physical performance and subjective health status regardless of previous ventilation [12].

We hypothesized that post-COVID-19 patients would benefit from PR as much as other patients with lung diseases. Thus, we compared the PR results, e.g., 6-min walk test (6-MWT), Feeling Thermometer (FT), and Functional Independence Measurement (FIM), of all post-COVID-19 patients to the outcome of patients with lung diseases referred to PR in 2019.

## 2. Materials and Methods

### 2.1. Participants and Procedures

All patients were referred for PR to the Zurich RehaCenter Klinik Wald, Switzerland after hospitalization between March and December 2020 following acute care phase of a COVID-19 infection. The data of these patients were prospectively analyzed according to performance and outcome during rehabilitation and were compared to the cohort of rehabilitation participants with different pulmonary diseases of the year 2019. Data of this patient group (lung diseases (LG)) were retrospectively analyzed to describe potential differences, performance, and outcome during PR of patients after severe COVID-19. In total, 92% of the patients of the control group participated in the PR program following severe exacerbation of their pulmonary disease requiring hospitalization. We used the German version of the program RehaTIS^TM^ by Softsolution, International AG, 15830 Lahti, Finland to record and control the individual rehabilitation process of each participant, including all therapies and procedures. The patient data and results of the assessments were stored and taken for evaluation out of the clinic information system Phoenix^TM^, CompuGroup Medical AG, 3007 Bern, Switzerland.

Post-COVID-19 patients were eligible for PR as soon as they were hemodynamically stable without the need of catecholamine or invasive ventilation and continuous monitoring. In the initial phase of the corona crisis in Switzerland, patients were admitted after being asymptomatic for 2 days and 10 days after onset of infection. Later on, patients were additionally required to have at least one negative swab before transfer, while between June and December, the swab strategy was dropped again, and the initial regulations were valid again. All patients gave written informed consent, and the local ethics committee approved the study protocol (BASEC-No 2020-01061). This study is registered at the German Clinical Trials Register (DRKS00024613).

Within two days after admission for rehabilitation, all patients from the COVID-19 cohort and PR cohort of 2019 were assessed with questionnaires, such as Chronic Respiratory Disease Questionnaire (CRQ), Hospital Anxiety and Depression Scale (HADS), and Cumulative Illness Rating scale (CIRS) and Functional Independence Measure (FIM). To measure changes during rehabilitation, 6-min walk test (6-MWT) and Feeling Thermometer (FT) were performed on admission and before discharge. All patients were deemed cognitively able to provide valid responses to the questionnaires by treating physicians. Comorbidities, pulmonary function testing (PFT), and laboratory values including blood gas analysis were assessed.

### 2.2. Pulmonary Rehabilitation

The standardized inpatient PR program had a duration of 3 weeks, including a total of 25–30 therapy sessions on 5–6 weekdays. The multimodal program mainly consisted of an individualized endurance exercise and strength training and was carried out according to a protocol adapted to the severity of the disease and functional physical limitations. Monday to Friday, patients participated in a maximum of 4 exercise sessions per day. On Saturday, one exercise session was offered, and Sunday was exercise-free. Exercise therapy consisted of endurance training (cycling and treadmill), gymnastics (3 levels), in- and outdoor walking (3 levels), and strength training.

For cycling exercise, a low-intensity interval program was chosen if the initial 6-MWT distance was <200 m. In this cohort, a walking distance of <200 m according to the initial 6-MWT was found in 58 patients. The duration of the higher and lower intensity intervals was set to 30–60 (55–70% of maximum heart rate) and 60 s, respectively. The rate of perceived exertion (RPE) measured by the adapted Borg Scale (1–10) was used to define and adapt exercise intensity with a goal of Borg 4–6 (dyspnea) during exercise [16,17,18]. Since most patients were very weak on PR admission, exercise started at very low intensity and increased continuously according to the patients’ tolerance. RPE was assessed after each exercise session. Duration of cycling was titrated individually starting at 5–10 min at entry and increasing over time up to 30–35 min at discharge. Exercise duration was usually increased first, with the goal to reach 20 min. After that, intensity was increased as well. All patients were monitored using pulse oximetry during their exercise. Criteria for stopping or reducing exercise intensity were an oxygen saturation (SpO_2_) < 88% and dyspnea symptoms (Borg ≥ 6). When a drop in SpO_2_ was observed, oxygen was added with a maximum of 6–8 L per minute via nasal cannula to keep the SpO_2_ > 90%. More than half of PG patients (*n* = 58) had an initial 6-MWT distance < 200 m, which required an individual adaptation of training. Partially bedridden patients started with in-bed cycling and MOTO-Med^®^ (RECK-Technik GmbH & Co. KG Medizintechnik, Reckstraße 1–5, D-88422 Betzenweiler, Germany). This was followed by first walking attempts with walking aids until first cycling interval training was possible. For fitter patients (6-MWT distance > 200 m), endurance training was carried out either on the treadmill or cycle ergometer. The speed of treadmill training was calculated based on the result of the 6-MWT (80% of 6-MWT pace; 6-MWT distance × 0.008 = exercise speed in km/h) but was also symptom-limited (Borg ≥ 6).

Outdoor walking was offered at different levels, with level 1 at a slow pace with little incline of the terrain and level 3 at a faster pace with frequent inclines. To decide which level of gymnastics or outdoor walking suited a patient, the result of the 6MWD was used (gymnastics: 6-MWT < 200 m = level 1, >400 = level 3) or an individual outdoor test walk was performed.

Gymnastics was offered in three levels of intensity. Whereas exercises in gymnastics level 1 took place mostly in a sitting position with several breaks between exercises, level 3 consisted of exercise in a standing position or walking with only very few or no breaks at all between exercises. Gymnastics consisted of a mixture of exercises to improve endurance, strength, coordination, range of motion, and balance.

A physio- or sports therapist instructed strength exercise 3–4 times per week individually according to recent American Thoracic Society/European Respiratory Society recommendations [17]. The modified Borg Scale was used to define exercise intensity with the goal of Borg 4–5. Usually, 3 series of 8–12 repetitions per exercise and 3–5 exercises for large muscle groups were chosen. In addition, patients received inspiratory muscle training and relaxation (progressive muscle relaxation).

Respiratory physiotherapy consisted of teaching breath control (pursed lip breathing, secretion mobilization, and diaphragmatic breathing), energy saving techniques, and controlled coughing exercises.

Twice a week (1 h each), all patients participated in educational sessions, including self-management, coping skills, self-medication, management of infections and exacerbations, dyspnea, use of oxygen, and nutrition interventions. If needed, patients took part in a structured smoking cessation program, received psychosocial support or diabetes advice. Table 1 provides an overview of the different therapies and their respective durations.

### 2.3. Hygiene Concept during Rehabilitation

For the treatment and management of COVID-19 patients, the recommendations of the Swiss Noso (Swiss National Institute of Infection-Prevention) and the Health Department of the Kanton Zurich were applied. The hygiene concept has been published recently [11,12]. An assessment of the hygienic risk was made for the individual patient. If isolation was indicated, the patients were transferred to the isolation ward where they were supervised in a single room. The frequency of these treatments was adapted to the individual and diagnosed limitations and needs of the patients. Due to the risk of infection, a ban on visits by relatives was implemented by the Health Department of the Kanton of Zurich for both the first and the second wave. This ban on visits was very stressful for the patients and in some cases also led to shortened stays in rehabilitation or even to direct discharge to their homes without rehabilitation.

### 2.4. Exercise Capacity

Exercise capacity was measured at hospital admission and discharge using the 6-min walk test (6-MWT), performed once at the beginning and once at the end of the PR program after 20 days, according to the guidelines of the American Thoracic Society and carried out by experienced, well-instructed examiners [19]. A change of 14.0 to 30.5 m was found to be clinically important across multiple patient groups [20]. Recently, for patients surviving acute respiratory failure or acute respiratory distress syndrome, a minimally important difference (MID) of 20 to 30 m was determined [21]. The median distance walked in the 6-MWT for healthy adults is described as 576 m for men and 494 m for women [22].

### 2.5. Quality of Life

As the standardized health-related quality of life (HRQoL) measurement tool, the German version of the Chronic Respiratory Disease Questionnaire (CRQ) was used. The questionnaire measures eight dimensions of HRQoL and allows calculation of two summary scales of physical and mental experienced [23].

### 2.6. Functional Independence Measure (FIM)

The FIM is an 18-item measurement tool that explores the severity of an individual’s physical and psychological disability, especially of rehabilitation patients. [24]. The tool is also used to assess a change in patients’ functional status in response to rehabilitation or medical intervention. The FIM uses the level of assistance an individual needs to grade functional status from total independence to total assistance. As the severity of disability changes during rehabilitation, the data generated by the FIM Instrument can be used to track such changes and analyze the outcomes of rehabilitation. FIM change scores associated with MID were 22 for the total FIM, motor FIM, and cognitive FIM, respectively [25].

### 2.7. Hospital Anxiety and Depression Scale (HADS)

The HADS was originally designed as a short, easy-to-use, 14-item screening tool for depression in patient status in response to rehabilitation. Both scales ranging from 0 to 21 with higher scores indicate more severe distress [26].

### 2.8. Cumulative Illness Rating Scale (CIRS)

We used the CIRS to assess a patient’s level of disability and as an indicator of health status, including predicted 18-month mortality and social function [27]. The calculated CIRS on admission is useful for predicting important hospital outcomes such as high risk of death or long stays and to better anticipate end of life issues.

### 2.9. Feeling Thermometer (FT)

We used the FT to determine and compare patients’ feelings about their actual wellbeing by applying a numeric rating of their feelings toward an imaginary scale in terms of degrees, with their attitudes corresponding to temperatures. The MID is defined between 5 and 8 degrees [28].

### 2.10. Pulmonary Function Tests (PFT) and Blood Gas Analysis

Spirometry and Body-Plethysmography (Master Screen Body; Jaeger GmbH, Hoechberg, Germany) were performed once on PR discharge in accordance with recent guidelines [29,30]. Arterial blood gases were taken at rest under room air condition (Radiometer ABL800, Willich, Germany) at the admission to PR [31]. Blood count, creatinine, and C-reactive protein (CRP) were obtained from the external laboratory Medica, Medizinische Laboratorien Dr. F. Kaeppeli AG, Zurich.

### 2.11. Statistics

Binary variables were presented as relative and absolute frequencies, and the Fisher’s exact test was used for group comparison. Normally distributed continuous variables were presented as mean with standard deviation (SD), and the t-test was used for comparison between groups. Not normally distributed continuous variables were presented as median with inter-quartile range (IQR), and the Mann–Whitney-U-Test was used for group comparison. For time-dependent comparisons, paired t-test was calculated. Boxplots were used as graphical presentation of the data. Linear models were calculated for multivariate analysis. *p*-value < 0.05 was considered as statistically significant. All analyses were conducted using Microsoft R Open 4.0.2, Microsoft Corporation, One Microsoft Way, Redmond, WA 98052-6399, USA.

## 3. Results

Between March and December 2020, 99 post-COVID-19 patients (PG) were referred to PR, and their results were compared to those of patients with different pulmonary diseases referred to PR in 2019 (*n* = 419) (LG).

### 3.1. Baseline Characteristics of the Post-COVID-19 Group (PG) and the Control Group (LG)

According to age, sex distribution, duration of PR, and CIRS, no significant differences were found between PG and LG. The results of the comparison of both groups are provided in Table 2. The BMI was significantly higher in the PG (28.21 vs. 24.5; *p* < 0.001). In the LG, COPD was the most common diagnosis (53%), followed by infectious pulmonary diseases (13%) and lung cancer (13%).

### 3.2. Comorbidities of the Post-COVID-19-Group (PG) and Complications Due to the COVID-19 Infection

The mean duration of the acute hospital stay of the PG was 25.9 (±8.81) days, with 11.3 (±12.5) days in the ICU and 7 (±9.18) days on ventilation. In total, 65 patients (66%) needed ICU care. On average, 2.9 comorbidities prior to the COVID-19 infection were present for PG patients with hypertension as the most prevalent comorbidity (54%). Table 3 provides the comorbidities of the PG.

Table 4 provides the complications due to the COVID-19 infection and the new diagnosis made during the hospital stay or during the rehabilitation indicating that the patients experienced on average 2.7 complications or new diagnosis.

### 3.3. Assessments on Admission or Discharge to PR of the Post-COVID-19 Group

Table 5 shows the results of the assessments and the laboratory parameters of the PG at admission to PR as well as the results of the Pulmonary Function Test. Chest X-rays at discharge from PR showed significant infiltrates in 69 (70%) of the PG. Oxygen supply at acute hospital discharge was necessary in 52 patients (53%), while oxygen supply at discharge from PR was needed in 25 patients (25%). Length of stay was 45.9 days (25.9 days in acute hospital and 20 days at PR) for the PG. Pulmonary Function Tests were available for 69 patients. A total of 12 patients (17%) had mild (*n* = 12; 17%), 28 patients (41%) moderate, and 11 patients severe (41%) restrictive ventilation pattern at the last third of PR. No restriction was found in 18 patients (26%). Diffusion capacity in the PG was found to be limited mild in 14 (20%), moderate in 28 (41%), and severe in 18 (26%) patients, while 9 (13%) patients had normal diffusion capacity.

### 3.4. Results of Pulmonary Rehabilitation in the PG and LG

**Figure 1:**
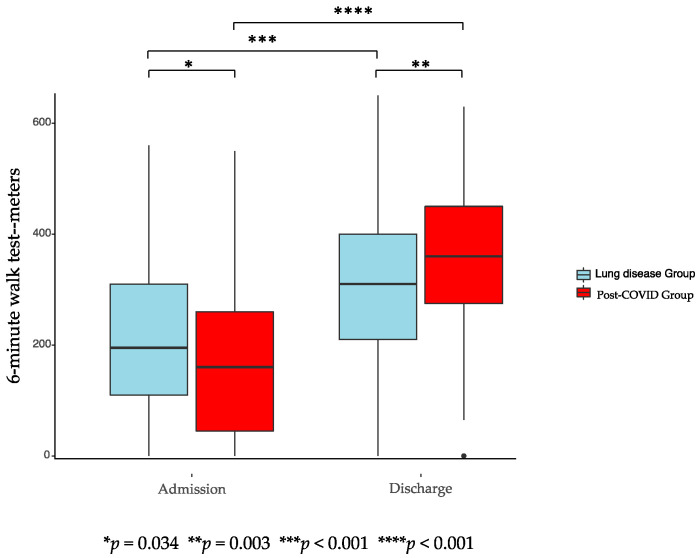
6-min walk test of post-COVID-19 Group and lung diseases group.

**Figure 2:**
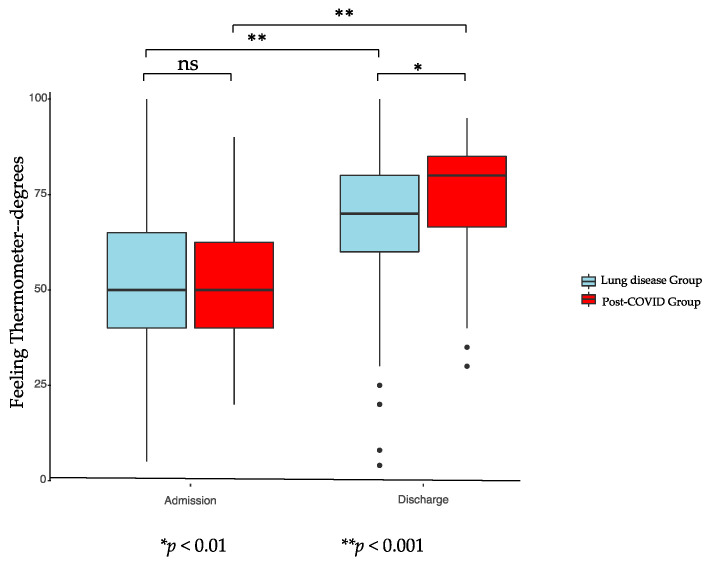
Feeling thermometer of post-COVID-19 group and lung diseases group. ns: no significative.

**Figure 3:**
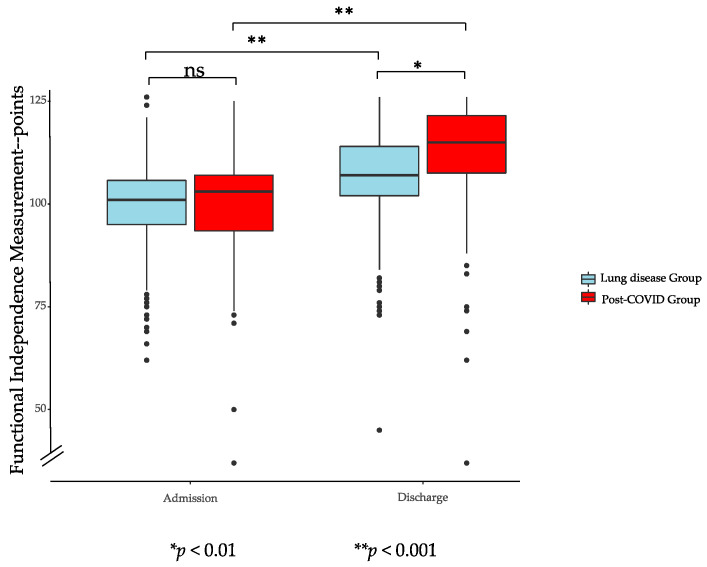
Functional Independence Measurement of post-COVID-19 group and lung diseases group. ns: no significative.

Figure 1, Figure 2 and Figure 3 show the results of the assessments at admission and discharge for both groups. PG and LG showed significant improvements during PR according to FIM, 6-MWT, and FT in the intragroup comparison. Improvements in the intergroup comparison were higher in the PG compared to the LG. The increase in the 6-MWT in the pre to post comparison were on average 180 (±101) meters for PG and 102 (±89) meters for LG (*p* < 0.001). FT showed a significant enhancement for PG of 21 (±14) points and for LG of 17 (±16) points (*p* < 0.039), while FIM significantly increased with 11 (±10) points in PG and 7 (±8) points in LG (*p* < 0.001).

Results of inter- and intragroup comparisons of FIM, 6-MWT, and FT on admission and discharge to PR are displayed in Table 6, showing significant improvements for all parameters in both groups in the intragroup comparison. While there were no significant differences found at discharge to PR for FT and FIM between both groups, the 6-MWT distance was significantly smaller for the PG. The increase for all parameters during PR was significantly higher for the PG in the intergroup comparison.

### 3.5. Multivariate Analysis of the Results

In order to prove results really depend on the group affiliation, a linear model was performed with the ∆ of FT, FIM, and 6-MWT as the dependent variable. Table 7 provides a multivariate analysis showing that the LG group affiliation has significant negative impact on the change in 6-MWT, FT, and FIM regardless of age, BMI, and sex.

## 4. Discussion

To the best of our knowledge, this is the first study comparing the results of PR of a larger group of severely impaired Post-COVID-19 patients to individuals usually referred to PR. Our results demonstrate that improvements during PR were significantly higher for the PG. This was not only true for the physical performance measured with FIM and 6-MWT but also for the actual wellbeing of the patients indicated by the results of the FT.

It is known that up to one third of COVID-19 patients developed severe pulmonary complications and acute respiratory distress syndrome (ARDS), leading to persistent impairments in pulmonary function and physical performance [2]. Indeed, the severity of impairments was more pronounced in patients with severe and critical COVID-19 courses [32]. Previously, we showed that patients with acute deteriorations of pulmonary function do benefit from PR [33]. However, little is known about the extent to which post-COVID-19 patients benefit from PR. The limitations in physical capacity according to the results of the initial 6-MWT of post-COVID-19 patients exceed the limitations observed in patients usually participating in PR. Nevertheless, post-COVID-19 patients did benefit to a greater extent from PR than LG. Indeed, our multivariate analysis clearly demonstrated that COVID-19 itself had a strong impact on changes in FT, FIM, and 6-MWT.

Despite the pronounced limitations, we suspect the cause of the significantly higher improvements to be the greater recovery potential of post-COVID patients compared with chronically ill patients, many of whom had long-standing pulmonary disease. Apparently, the pre-existing and acquired comorbidities in the context of COVID-19 infection were comparable to the lung diseases group, yet the potential for improvement was higher in the PG. We suspect that the acquired physical and psychological limitations in the PG were predominantly at least partially reversible, which was obviously not the case in the LG. However, our results are in line with a recent publication reporting significant improvements in performance status and lung function of COVID-19 patients by a structured multidisciplinary rehabilitation program [34].

Despite the significant improvements in our COVID-19 cohort, chest X-rays, spirometry, body plethysmography, and lung diffusion capacity (DLCO) were still impaired even at the end of PR. These results are in line with the study of Puchner et al. showing that 57% of all COVID-19 patients had pathological FEV1, FVC, and/or TLC < 80%, and 83% of all study participants still had a reduction of the DLCO at the end of the rehabilitation program [34]. Compared to Puchner et al., our COVID-19 patients showed significantly higher physical limitations in 6-MWT on PR admission; however, the absolute improvements during PR were identical. The observed differences in 6-MWT cannot be explained by impaired lung function alone since available data of lung function of both studies are comparable.

The initially very strongly elevated CRP values of PG patients indicate a still present inflammatory load associated with disease severity and can partly explain patients’ limitations. In this context, it is of note that lengths of stay in rehabilitation of COVID-19 patients were comparable to those of normal PR patients, despite persisting limitations. Several aspects can be taken into account for identical PR duration. One reason is the long absence from the home environment. On average, patients were away from home for more than 45 days, which many patients reported to be enormously stressful. In addition, a ban on visits to the clinic had to be imposed due to the pandemic, which meant that communication with relatives was only possible via phone calls.

Although several patients still showed significant limitations before discharge, no patient from our cohort had to be referred to an institution following PR. After participation in PR, further care of the patients was possible in the home environment with the support of walking aids, home care, the help of relatives or oxygen supplementation. These results are in contrast to the findings of Roberts et al. showing that physical function deficits in COVID-19 patients were associated with an increased odds ratio of discharge to an institution from acute care hospitals [7]. In Switzerland, patients with severe COVID-19 infection and persistent significant functional deficits were given the option to be transferred to an inpatient rehabilitation program for post-acute treatment. Fortunately, the costs for inpatient rehabilitation are covered by the Swiss health system. For this reason, PR in the post-acute phase of severe COVID-19 infection represents an important link in the care chain.

### Study Limitations

We have to acknowledge that the present study has some limitations. First, due to the observational design of the study, we cannot report on the causality of the observed findings. COVID-19 specific findings cannot be claimed without an appropriate COVID-19 control group not referred to PR. The comparison group used in this study is only conditionally suitable to make an adequate comparison. Due to the retrospective collection of data and the resulting missing data of the control group, direct comparison regarding, e.g., PFT, HADS, CRQ or comorbidities has not been possible. Second, no statement can be made about the impact of specific treatment measures since this study was rather focused on the overall effects of the multidisciplinary treatment plan. Thus, we cannot differentiate the influencing value of specific treatment sessions on the overall outcome of the rehabilitation plan. Finally, this study only provides data for the immediate PR effect; long-term results cannot be derived from this. Long-term monitoring of further PR needs, reporting of quality of life, and long-term benefits of rehabilitation must be provided by future studies.

## 5. Conclusions

Our study provides evidence that post-acute comprehensive pulmonary rehabilitation is associated with significant clinical and functional improvements in individuals who suffered from severe COVID-19 and underlines the importance of post-acute rehabilitation for COVID-19 recovery. Consequently, healthcare facilities should develop and implement plans for providing multidisciplinary rehabilitation treatments in various settings to recover functioning and prevent the development of long-term consequences of the COVID-19 disease.

## Figures and Tables

**Table 1:** Available exercise frequency and minutes of therapies.

Type of Exercise	Frequency per Week	Maximum Duration per Session (min)
Endurance	5–6	10–30
Gymnastics	5–6	45
Outdoor walking	2–3	45
Strength training	3–4	30
Relaxation	2	45
Respiratory therapy	3	30

**Table 2:** Comparison of the post-COVID-19 group (PG) to the lung diseases group (LG).

	PG (*n* = 99)	LG (*n* = 419)	*p*
Age (mean)	67.72 (±10.23)	69.28 (±11.29)	0.207
Sex, female (%)	42 (±42.4)	213 (±50.8)	0.163
BMI kg/m2 (mean)	28.21 (±6.11)	24.50 (±6.10)	<0.001
PR days (median [IQR])	20 [18.00, 22.00]	21 [18.00, 21.00]	0.042
CIRS points (mean)	14.18 (±5.92)	14.52 (±5.6)	0.324

Notes: PG, post-COVID-Group; LG, lung diseases group; BMI, body mass index; PR, pulmonary rehabilitation; CIRS, Cumulative Illness Rating Scale.

**Table 3:** Comorbidities of post-COVID-19 patients prior to COVID-19 infection.

Comorbidities Prior COVID-19	
(*n* = 99)	*n* (%)
Hypertension	54 (54)
Smokers or Ex-Smokers	27 (27)
Adiposities	25 (25)
Musculoskeletal disease	25 (25)
Dyslipidemia	20 (20.2)
Neurological disease	20 (20)
Chronic renal failure	19 (19)
Coronary artery disease	18 (18.2)
Malignancy	15 (15)
COPD	11 (11)
Cerebrovascular insufficiency	9 (9)
Atrial Fibrillation	8 (8.1)
Diabetes	8 (8)
Obstructive sleep apnea	7 (7)
Chronic heart failure	6 (6)
Venous thromboembolism	5 (5)
Interstitial lung disease	5 (5)
Liver disease	5 (5)

Notes: ICU, intensive care unit; COPD, chronic obstructive pulmonary disease.

**Table 4:** Complications due to COVID-19 infection or new diagnosis made during inpatient stay.

Complications or New Diagnosis	*n* (%)
Sepsis	37 (37)
Delirium	36 (35)
ARDS severe	27 (27)
ICU acquired weakness	24 (24)
Anemia	24 (24)
Electrolyte disturbance	18 (18)
Acute renal failure	14 (14)
Atrial Fibrillation	13 (13)
Myocarditis	12 (12)
Hepatitis	12 (12)
Venous Thromboembolism	11 (11)
Bacterial superinfection	11 (11)
Diabetes	8 (8)
Arterial Hypertension	6 (6)
Acute Heart failure	4 (2)
ARDS mild	4 (4)
COPD	1 (1)
Coronary Artery Disease	1 (1)
Pulmonal Artery Disease	1 (1)

Notes: ARDS, acute respiratory distress syndrome; ICU, intensive care unit; COPD, chronic obstructive pulmonary disease.

**Table 5:** Results of the assessments at admission to PR of the post-COVID-19 Group (PG).

	Mean	SD
Assessments on admission to PR (*n* = 99)		
CIRS; points	14.2	5.8
HADS A; points	5.58	3.87
HADS D; points	5.52	3.09
FIM total; points	100	15.1
FIM social; points	29	4,65
FIM motoric; points	71.4	12.3
CRQ; points	4.66	0.93
Laboratory parameters on admission to PR		
PaO_2_ (kPa) (*n* = 72)	9.22	1.85
PaCO_2_ (kPa) (*n* = 72)	4.6	0.68
SpO_2_% (*n* = 99)	93.6	3
C-Reactive Protein (mg/dL) (*n* = 88)	151	134
Ferritin (mg/L) (*n* = 55)	1190	1170
Hemoglobin (mg/L) (*n* = 99)	99.8	24.1
Creatinin (mg/L) (*n* = 99)	128	122
Pulmonary function test at discharge PR (*n* = 69)		
FEV1 % pred.	74.9	38.94
FVC % pred.	74.1	37.6
FEV1 % FVC	85.2	25.6
DLCO % pred.	61	38.56

Notes: CIRS, Cumulative Illness Rating Scale; HADS A, Hospital Anxiety and Depression Scale Anxiety; HADS D, Hospital Anxiety and Depression Scale Depression; FIM, Functional Independence Measurement; CRQ, Chronic Respiratory Questionnaire; FEV1, forced expiratory volume in one second; FVC, forced vital capacity; DLCO, lung diffusion capacity.

**Table 6:** Inter- and intragroup comparisons of FT, FIM and 6-MWT on admission and discharge to PR.

	LG			PG			Intergroup	
	Pre	Post	*p*	Pre	Post	*p*	*p* Pre	*p* Post
FIM (points)	99.7 (±9.72)	107 (±10.7)	<0.0001	100 (±15.1)	111 (±15.0)	<0.0001	0.771	0.0063
6-MWT (meter)	210 (±128)	312 (±126)	<0.0001	176 (±141)	357 (±132)	<0.0001	0.034	0.0026
FT (degrees)	51.9 (±18.1)	68.6 (±17.2)	<0.0001	52.6 (±15.5)	73.8 (±14.5)	<0.0001	0.706	0.0038

Notes: FIM, Functional Independence Measurement; 6-MWT, 6-min walk test; FT, feeling thermometer.

**Table 7:** Multivariate analysis with the ∆ of the FT, the FIM and the 6-MWT as the dependent variable.

	Δ FT		Δ FIM		Δ 6-MWT	
	Beta [95% CI]	*p*	Beta [95% CI]	*p*	Beta [95% CI]	*p*
(Intercept)	20.09 [6.32–33.87]	0.004	9.49 [2.43–16.55]	0.009	237.47 [172.84–302.09]	<0.001
No Covid (LG)	−4.37 [−8.23–−0.52]	0.026	−3.62 [−5.69–−1.56]	0.001	−81.03 [−101.78–−60.27]	<0.001
Age	0.06 [−0.10–0.21]	0.477	0.00 [−0.08–0.07]	0.933	−0.32 [−1.04–0.39]	0.374
Sex, female	0.00 [−3.36–3.36]	0.999	−1.40 [−3.10–0.30]	0.105	−11.55 [−27.38–4.28]	0.153
BMI	−0.09 [−0.37–0.19]	0.526	0.08 [−0.05–0.22]	0.232	−1.06 [−2.36–0.24]	0.108

Notes: FT, Feeling Thermometer; FIM, Functional Independence Measurement; 6-MWT, Six-Minute Walk Test; BMI, body mass index.

## Data Availability

Data supporting the reported results can be provided upon reasonable request by the corresponding author.
